# Report of Three Cases of Dislocation of the Femur Reduced by Manipulation

**Published:** 1856-03

**Authors:** 


*Report of Three Cases of Dislocation of the Femur Reduced by Manipulation. New Method of Reducing Dislocations of the Femur on the Pubes.* By E. J. Fountain, M. D., of Davenport, Iowa.

Recently I sent to Dr. Reid, of Rochester, New York, a brief report of three cases of dislocation of the thigh,—one upon dislocation of the dorsum ilii, and two upon the pubis,—all reduced by manipulation. At his suggestion I send a full report of these cases for publication. The case of dislocation upon the dorsum was reduced very quickly and easily by following exactly the directions given by Dr. Reid, with whom this vastly improved method originated. The two dislocations upon the pubis, I reduced by manipulations based upon the same principles; but by a mode of manipulating quite different from that required for the reduction of a dislocation upon the dorsum ilii. The report of these two cases will be the first of the kind upon record. A concise summary of the rules for the operation will be appended to the report.

*Dislocation upon the Dorsum Ilii.*—Oct. 7th. I was called in the night to go in haste to the relief of a lady, Mrs. S——, who had received, as stated, some serious injury of the hip, or thigh, by being thrown from a wagon. I was accompanied by my partner, Dr. Adler. We found the patient in bed, complaining of pain in the left hip. The examination revealed at once the nature of the injury, which was a dislocation of the left femur upon the dorsum ilii. On placing the patient erect, the characteristic appearance was presented. The knee rested upon the lower third of the thigh, the great toe of the foot upon the instep of the opposite limb, and the trochanter major approximated to the crest of the ilium. The diagnosis was confirmed by an attempt to rotate and abduct the limb. A matress was thrown upon the floor, and upon this the patient was placed upon her back. A towel was carried around the sound thigh and hip, and held down by Dr. Adler; but this assistance I found to be quite unnecessary. I then grasped the knee with my right hand, and the foot with my left; flexed the leg on the thigh, and carried the knee and thigh over and upon the sound one, and then upward as high as the umbilicus, keeping it constantly pressed down upon the body. I then carried the knee outward, bringing the heel inward and the foot over the opposite limb, at the same time making gentle oscillations of the thigh, when the head of the bone slipped suddenly into its socket. The force required was quite moderate, and the pain almost nothing. The *time* occupied by the manipulation, from the instant I took hold of the knee and foot, until the operation was completed, did not exceed *ten seconds.* The manipulations were made by one continuous uninterrupted motion. The knee was caused to make a "semi-circular sweep" over the sound limb and across the body, then a few quick oscillations, and it dropped down into its natural position. I held the thigh up firmly and steadily, while making the oscillations; and in this position, at right angles with the axis of the body, and abducted, and the foot over the opposite thigh, the head of the bone entered its socket.

*Dislocations on the Pubis.* Case I.—In June, 1854, I was called to see a man who had fallen from the second story of a house to the ground, upon some pieces of timber. His lower jaw was fractured, and his left hip dislocated. The limb was a trifle shortened, and the foot strongly everted. The prominence of the trochanter major was lessened, and the head of the bone could be felt upon the pubis. While waiting for the appearance of Dr. Arnold, who had also been sent for, I was reflecting upon the necessary arrangements to be made for the application of the *pullies.* While thus meditating upon the subject, I began to think of the possibility of reducing the dislocation by manipulation. Considering the position of the head of the bone and the relation to adjacent parts, it occurred to me that by rotating the limb still more strongly outward, I might elevate the head of the bone from its resting-place—the trochanter major acting as a fulcrum. Then, by carrying the leg and foot, and after it the knee and thigh, over the opposite thigh, while the limb was still strongly rotated outward, the head of the bone would be made to move upward and outward in the arc of a circle of which the trochanter major would be the center, and the neck the radius. After being thus brought over and upon the edge of the acetabulum, a motion of the limb directly upward, would, in the same way, throw the head of the bone into its socket—the muscles attached to the trochanter major holding that point comparatively fixed.

Before the arrival of Dr. Arnold, I had determined to test the theory; and on explaining my views to him, he at once expressed his willingness to have the attempt made as I suggested. At worst it could only fail without much if any harm, and then we had the pullies ready for application after the "classical method."

The patient was placed upon the floor on a quilt. Being a man of strong muscular development, I thought there would be more certainty of success if relaxation was first produced by the inhalation of chloroform. He readily came under its influence. When quite unconscious, the limb was taken by the foot and knee and rotated outward, the leg flexed and carried over the opposite knee and thigh, the heel kept well up and the knee pressed down. This motion was continued by carrying the thigh over the sound one as high as the upper part of the middle third, the foot kept firmly elevated. Then the limb was carried directly upward by elevating the knee, while the foot was held firm and steady, at the same time making gentle oscillations by the iknee, when the head of the bone suddenly dropped into its socket.

Time required in the operation, from twenty to thirty seconds. The force used was slight. I believe it could have been reduced about as well without the chloroform.

Case II.—Oct. 31st, 1855. John McCarthy, an Irishman, had his hip dislocated by falling with a horse he was riding. The horse slipped and fell, rolling over upon him. I found the limb about the same in length as the sound one; but greatly everted, the toes pointing directly outward. On attempting to rotate and flex the limb, pain was produced, and a comparative immobility manifested by resistance. The head of the bone was felt forward upon the pubes. As soon as I discovered it was a dislocation, my first thought was to send for Dr. Adler to witness the operation. But the temptation to take hold and reduce it immediately, was too strong. The patient was resting upon a low couch. I immediately took hold of his knee and foot, rotated outward, and flexed the leg by carrying the foot over the sound thigh, keeping the heel well up and pressing the knee down. After I had brought the thigh in this way over the upper part of the sound one, I carried it directly upward, holding the foot firmly up and making oscillations by the knee, when the head of the bone slipped into its socket, and the limb at once assumed its natural appearance and mobility. A little more force was required in this than in the other case; but it was still quite moderate, and the pain very slight. In this case I had no assistance whatever. Time occupied in operating, about twenty seconds.

The history of these cases fully demonstrates, to my mind, the immense value of this new method of reducing dislocations of the hip. Notwithstanding the unsatisfactory results of the trials at the New York Hospital, I have perfect confidence in the correctness of Dr. Reid's method of manipulation. It is certainly one of the greatest improvements of modern surgery, the value of which may be understood, when contrasting an operation requiring but ten or twenty seconds, and without pain, with the instructions of Sir Astley Cooper, viz: "Venesection to syncope, hot bath, tart. antimony to nausea, and then the application of the pullies from four to six hours, if necessary." To Dr. Reid is due the credit of this splendid improvement, in which the whole profession must participate, as a most valuable contribution of scientific surgery to the relief of suffering humanity. It remains to be seen how far the test of future operations will confirm the value and correctness of the method of reducing dislocations on the *pubis,* as illustrated by the two preceding cases.

It is my opinion that dislocations into the thyroid foramen may be reduced by the same method as the last.

In conclusion, I will recapitulate the method of operating for dislocations on the *pubis.*

Taking the knee in one hand and the foot in the other, rotate the whole limb outward, and flex the leg on the thigh by carrying the foot over the opposite knee. Then carry the limb, foot forward, over the opposite thigh, at the same time twisting the heel upward, and pressing the knee down. Carry the thigh in this way over the sound one, as high as the upper part of its middle third; then elevate the limb by raising the knee while the foot is held firm, at the same time making gentle oscillations, when the head of the bone will slip suddenly into its socket.—*N. Y. Jour. of Med.*

